# Depletion of the Third Complement Component Ameliorates Age-Dependent Oxidative Stress and Positively Modulates Autophagic Activity in Aged Retinas in a Mouse Model

**DOI:** 10.1155/2017/5306790

**Published:** 2017-08-08

**Authors:** Dorota Rogińska, Miłosz P. Kawa, Ewa Pius-Sadowska, Renata Lejkowska, Karolina Łuczkowska, Barbara Wiszniewska, Kai Kaarniranta, Jussi J. Paterno, Christian A. Schmidt, Bogusław Machaliński, Anna Machalińska

**Affiliations:** ^1^Department of General Pathology, Pomeranian Medical University, Al. Powstancow Wlkp. 72, 70-111 Szczecin, Poland; ^2^Department of Histology and Embryology, Pomeranian Medical University, Al. Powstancow Wlkp. 72, 70-111 Szczecin, Poland; ^3^Department of Ophthalmology, Institute of Clinical Medicine, University of Eastern Finland, 70211 Kuopio, Finland; ^4^Department of Ophthalmology, Kuopio University Hospital, 70211 Kuopio, Finland; ^5^Clinic for Internal Medicine C, University of Greifswald, 17475 Greifswald, Germany; ^6^Department of Ophthalmology, Pomeranian Medical University, Al. Powstancow Wlkp. 72, 70-111 Szczecin, Poland

## Abstract

The aim of the study was to investigate the influence of complement component C3 global depletion on the biological structure and function of the aged retina. In vivo morphology (OCT), electrophysiological function (ERG), and the expression of selected oxidative stress-, apoptosis-, and autophagy-related proteins were assessed in retinas of 12-month-old C3-deficient and WT mice. Moreover, global gene expression in retinas was analyzed by RNA arrays. We found that the absence of active C3 was associated with (1) alleviation of the age-dependent decrease in retinal thickness and gradual deterioration of retinal bioelectrical function, (2) significantly higher levels of antioxidant enzymes (catalase and glutathione reductase) and the antiapoptotic survivin and Mcl-1/Bak dimer, (3) lower expression of the cellular oxidative stress marker—4HNE—and decreased activity of proapoptotic caspase-3, (4) ameliorated retinal autophagic activity with localization of ubiquitinated protein conjugates commonly along the retinal pigment epithelium (RPE) layer, and (5) significantly increased expression of several gene sets associated with maintenance of the physiological functions of the neural retina. Our findings shed light on mechanisms of age-related retinal alterations by identifying C3 as a potential therapeutic target for retinal aging.

## 1. Introduction

Age-related deterioration of the retina seriously constrains the vision quality of a growing number of elderly people worldwide. Recently, the dysregulation of inflammatory and immune pathways has become increasingly accepted as key fundamental mechanisms of age-related retinal alterations [1].

It is widely accepted that age-related retinal cell injury caused by accumulative oxidative stress (OS) represents an initial determinant for various age-related retinal malfunctions. Lipofuscin, the aging-associated pigmented material, which accumulates in the cells of the retinal pigment epithelium (RPE) with age together with the accumulation of basal laminar deposits, and drusen in Bruch's membrane have been proposed to be mainly responsible for the increase in local oxidative stress in the retina [2]. Oxidative stress-related cellular products develop as a consequence of autophagy dysregulation in RPE cells. A large amount of evidence indicates that autophagy declines with age and that this progressive reduction may have a causative role in the development of age-related retinal alterations [3]. Impaired autophagy in the RPE leads to cell transcytosis and exocytosis and early signs of retinal degeneration [4]. Abnormal autophagic and ubiquitin-proteasome pathway (UPP) cleansing has been documented in aged RPE cells and in retinas from AMD patients [3–5].

Insufficient degradation of toxic proteins and oxidative lipids triggers an immune response in the affected tissues. In the elderly, the combination of increased production of reactive oxygen species (ROS) and decreased antioxidant functions, accompanied by an upregulation of several inflammatory genes, such as those encoding interleukin- (IL-) 1*β*, IL-6, IL-8, and tumor necrosis factor (TNF), leads to a bidirectional attack, both from the altered redox status and from the dysregulated immune responses [6]. Compelling evidence suggests that the complement system (CS) plays a critical role in regulating inflammatory and immunological processes in aged retinas. In humans, it was found that C3 gene expression is upregulated with aging [7]. Likewise, aged CD59-deficient animals exhibit increased expression of several activators of the alternative CS pathway (C3, CFB, and CFD), particularly in the RPE-choroid layer [8]. Thus, dysregulation of the complement system has been implicated in the pathogenesis of age-related retinal disorders, for example, AMD. Consequently, chronic and repetitive nonlethal RPE injuries, together with an oxidative environment and autophagy dysregulation, appear to be important factors for the development of age-related retinal changes.

Although there is a significant body of research on the mechanisms of retinal aging, the complex pathways between oxidative stress, initial cellular damage, locally triggered inflammation, autophagy, and UPP conditions are not yet apparent. Therefore, the aims of this study were twofold: (1) to investigate the effect of C3 depletion on oxidative metabolism, apoptosis, and autophagic and proteasomal cleansing in aged retinal tissue in vivo and (2) to investigate the effect of C3 deficiency on global gene expression in aging retinal tissue.

## 2. Materials and Methods

### 2.1. Animals

Pathogen-free 12-week-old mature C3-deficient (C3−/−) female mice (strain: B6;129S4-C3tm1Crr/J; Jackson Laboratory, Bar Harbor, ME, USA) and age-matched WT C57BL/6 mice (Polish Academy of Sciences, Wroclaw, Poland) were purchased and maintained under standard conditions with a 12-hour day (160-lux)-night cycle and with ad libitum feeding. These C3−/− mice produce no detectable C3 protein and no complement activity. Wild-type age- and gender-matched C57BL/6 littermates were used as the C3-positive controls. Each mouse strain was divided into two groups, and one group was maintained to an older age of 12 months. These animals were then used in the indicated experiments. All animal procedures were performed according to the regulations in the ARVO Statement for the Use of Animals in Ophthalmic and Vision Research and were approved by the local ethics committee. We followed the methods of Machalińska et al. [9].

### 2.2. Electroretinography

Scotopic and photopic ERGs were recorded from 3- and 12-month-old C3-deficient and WT mice (*n* = 10 mice, for each group of animals). Following overnight dark adaptation, the mice were anesthetized by an intraperitoneal injection of ketamine (40 mg/kg) and xylazine (4 mg/kg) (both from Biowet, Puławy, Poland). Then, the cornea was anesthetized (Alcaine; Alcon, Fort Worth, TX, USA), and the pupils were dilated with 1% atropine (Polfa Warszawa, Poland). Retinal responses were recorded with gold ring contact electrodes (LKC Technologies, Gaithersburg, MD, USA). Needle electrodes were placed under the scalp between the eyes and in the tail as the reference and ground leads, respectively. ERGs were differentially amplified (0.05–1500 Hz), averaged, and stored using an LKC UTAS BigShot system. ERGs were recorded in response to strobe flash stimuli presented in the LKC Ganzfeld bowl using a protocol similar to that used for human testing. To assess rod photoreceptor function, a strobe white-flash stimulus was presented to the dark-adapted dilated eye with a low flash intensity (24 dB attenuation), and 8 responses recorded at intervals of 8 s were computer-averaged. Mixed rod and cone responses were obtained by stimulation with white flashes of maximum intensity equal to approximately 1.6 cd^∗^s/m^2^ (standard flash, SF; 0 dB attenuation). The retinal responses were measured twice with a 28 s interstimulus interval and averaged. To evaluate the function of the cone photoreceptors, the animals were light-adapted for 10 min under a white background (32 cd/m^2^). Next, a strobe white-flash stimulus was presented to the dilated eye in the Ganzfeld bowl using the maximum flash intensity (0 dB attenuation), and responses to 8 flashes with an interstimulus interval of 1 s were recorded and averaged. The amplitude of the b-wave was measured from the a-wave trough to the peak of the b-wave or, if an a-wave was not present, from the prestimulus baseline to the peak of the b-wave. To demonstrate changes in the retinal bioelectrical response over time, the data for each individual 1-year-old mouse were calculated as a percentage value in reference to the b-wave amplitude measurements obtained from 3-month-old mice (which was considered 100%).

### 2.3. SD-OCT Imaging

Spectral domain optical coherence tomography was performed in 12-week-old and 12-month-old C3-deficient and WT mice (*n* = 10 mice, for both groups of animals). For the imaging procedures, the mice were anesthetized by intraperitoneal injection of ketamine (40 mg/kg) and xylazine (4 mg/kg). The pupils were dilated with 1% atropine. Artificial tears were used throughout the procedure to maintain corneal clarity. Images were obtained using the Envisu R2200-HR SD-OCT device (Bioptigen Inc., Durham, NC, USA) with the reference arm placed at approximately 1197 mm. SD-OCT images of a specific region of each eye were obtained using the optic disc as a landmark. Rectangular scans (1.5 mm × 1.5 mm, 1280 A-scans/B-scan × 150 B-scans) were obtained first while centering on the optic nerve and then with the nerve displaced either temporally/nasally or superiorly/inferiorly. For quantitative analysis, the distance between the first high reflective layer (i.e., the vitreoretinal interface) and the retinal pigment epithelium was measured using InVivoVue software. If the optic nerve was placed temporally/nasally, three B-scans (at the level of the nerve, 450 *μ*m up and down from the nerve) were added with one measurement performed 750 *μ*m away from the optic disc, on each side. When the optic nerve was placed superiorly/inferiorly, one B-scan was placed 750 *μ*m away from the optic disc with 3 measurements (at the level of the nerve, 450 *μ*m to the left and right of the nerve) for both positions. The mean value of all measurements was considered the total retinal thickness. To demonstrate changes in the total thickness of the retina over time, the data are presented as a percentage value calculated for each individual 1-year-old mouse in reference to measurements obtained from 3-month-old mice (which was considered 100%).

### 2.4. Tissue Collection

At the end of the experiment, 12-month-old C3-deficient and WT mice were sacrificed by cervical dislocation under anesthesia. The eyes were enucleated and divided for different assessments. For histological and immunofluorescence analyses, the tissue was fixed in Davidson's solution, embedded in paraffin, and cut into 5 *μ*m thick sections (*n* = 3 eyes in each group of animals). For Affymetrix Gene Chip Microarray, Western blot, and Luminex analyses, total RNA and protein were isolated using the PARIS Kit (Thermo Fisher Scientific, Waltham, MA, USA) following the manufacturer's instructions (*n* = 14 eyes in each group of animals).

### 2.5. Histological and Immunofluorescence Analyses

For cross sections, the tissue was routinely stained with hematoxylin and eosin (Sigma-Aldrich, Saint Louis, MO, USA). For immunofluorescence analysis, sections were deparaffinized in xylene (2 × 15 min) followed by hydration in solutions with decreasing ethanol concentrations (100, 95, 80, 75, and 50%). In the first stage of ubiquitin detection, slides were quenched with 0.1 M glycine in TBS for 10 minutes. After 30 minutes of blocking in IHC blocking solution (Bethyl Laboratories Inc., Montgomery, TX, USA), the sections were incubated with the primary antibody, rabbit anti-ubiquitin (1 : 200) (Dako Corporation, Carpinteria, CA, USA, cat. number Z0458), at 4°C overnight. On the next day, the secondary antibody, goat anti-rabbit-Alexa Fluor-488 (1 : 500), was applied followed by DAPI (dilution 1 : 10000) for nuclear staining. For the negative control sections, incubation with the primary antibody was omitted and the sections were stained only with the secondary antibody and DAPI solution. For C3 staining, Tyramide Signal Amplification (TSA) (Thermo Fisher Scientific, Waltham, MA, USA, cat. number T20925) protocol was applied, according to the manufacturer's instructions. Briefly, after 45 minutes of quenching with 3% H_2_O_2_ in PBS and 30 minutes of blocking in 1% BSA, the sections were incubated with the primary antibody, rabbit anti-C3 (1 : 100) (Bioss Antibodies Inc., Woburn, MA, USA, cat. number bs-2934R), at 4°C overnight. On the next day, the secondary antibody, goat anti-rabbit-HRP (1 : 100), was applied. Fluorescence detection and signal amplification were achieved by staining the sections with Tyramide-Alexa Fluor 594 in amplification buffer/0.0015% H_2_O_2_ for 6 minutes, at room temperature. DAPI was used for counterstain (1 : 10000). The sections from 12-month-old C3-deficient animals served as a staining control. After Mowiol mounting (Calbiochem, San Diego, CA, USA), all sections were subjected to fluorescence microscopy analysis (Carl Zeiss MicroImaging, Oberkochen, Germany).

### 2.6. Western Blot and Densitometry Analyses

Equal amounts of proteins (20 *μ*g/well) were loaded and separated by 4–20% sodium dodecyl sulfate polyacrylamide gel electrophoresis (SDS-PAGE, mini-PROTEAN II electrophoresis system, Bio-Rad, Hercules, CA, USA) and then transferred to a 0.2 *μ*m polyvinylidene fluoride (PVDF) membrane (Bio-Rad, Hercules, CA, USA). After blocking with 3% BSA in TBST, the membrane was probed with a primary antibody as follows: rabbit anti-catalase (1 : 750) (Abcam, Cambridge, UK, cat. number ab16731), rabbit anti-glutathione reductase (GSR) (1 : 1000) (Abcam, cat number ab16801), mouse anti-4HNE (1 : 1000) (Abcam, cat. number ab48506), rabbit anti-LC3B (1 : 375) (Abcam, cat. number ab48394), mouse anti-p62 (1 : 500) (Abcam, cat number ab56416), rabbit anti-ubiquitin (1 : 1000) (Dako Corporation, Carpinteria, CA, USA, cat. number Z0458), or mouse anti-*β*-actin (1 : 1200) (Santa Cruz Biotechnology, Dallas, TE, USA, cat. number sc-47778), and incubated overnight at 4°C. Immunoreactive bands were detected using a horseradish peroxidase-conjugated goat anti-rabbit and goat anti-mouse secondary antibody. Chemiluminescence detection was performed using the ECL Select Detection Kit (GE Healthcare, formerly Amersham Life Sciences, Little Chalfont, UK), and the bands were subsequently visualized using a UVP camera (GelDoc-It Imaging System; Bio-Rad, Hercules, CA, USA). Equal loading in the lanes was evaluated by stripping the blots for 2 h at 37°C and then overnight at room temperature (IgG Elution Buffer; Thermo Fisher Scientific, Waltham, MA, USA). Reprobing was then performed in an analogous manner with a mouse anti-*β*-actin as described above. The Band Analysis tool available in the ImageLab software version 4.1 was used to determine the background-subtracted density of the bands. The relative protein expression levels of 4HNE (4-hydroksy-2,3-nonenal), catalase, GSR, p62, and ubiquitin were quantified in comparison to those of *β*-actin. The LC3B relative protein expression level was calculated by estimation of the density of two bands, LC3B-I and LC3B-II, and presented as the LC3B-II/LC3B-I ratio.

### 2.7. Luminex Assay

Survivin, Mcl-1/Bak dimer, and active caspase-3 concentrations were quantified in C3-deficient and WT mouse retina homogenates by multiplex fluorescent bead-based immunoassays (Luminex Corporation, Austin, TX, USA) using commercial Bio-Plex Pro™ RBM Apoptosis Multiplex Assays, Panel 3 Analytes (Bio-Rad, Hercules, CA, USA). After adding blocking buffer to all wells of the plate, 30 *μ*L of each standard control and sample was added to the plate together with the multiplex antibody capture bead solution, and the plate was incubated with agitation for 1 hr at room temperature. Subsequently, each well was washed 3 times with 100 *μ*L assay buffer using a hand-held magnet. Next, 40 *μ*L of detection antibody cocktail was pipetted into each well, and the plate was sealed and incubated at room temperature for 1 hr on a shaker. After this step, 20 *μ*L streptavidin-phycoerythrin mixture was added to the plate which was incubated with agitation for 30 minutes in the dark. Finally, after washing, the microspheres in each well were resuspended in 100 *μ*L assay buffer and shaken at room temperature for 30 sec. The plate was then read and analyzed on the Luminex analyzer, and the analyte concentrations were determined from five different standard curves showing the MFI (median fluorescence intensity) versus protein concentration. To standardize the final concentration values, the obtained data were normalized to the total protein concentration.

### 2.8. Affymetrix GeneChip Microarray and Data Analysis

Total RNA isolated from four retinas obtained from 12-month-old C3-deficient and WT mice were pooled to generate two samples for the subsequent experimental procedures. A sense-strand cDNA generated from the total RNA using an Ambion WT Expression Kit (Thermo Fisher Scientific, Waltham, MA, USA) was subjected to fragmentation and labeling using the GeneChip WT Terminal Labeling Kit (Affymetrix, Santa Clara, CA) and then hybridized onto an Affymetrix Mouse Gene 2.1 ST Array Strip. Hybridization and subsequent fluidics and scanning steps were performed with an Affymetrix GeneAtlas™ System. The preliminary analysis of the scanned chips was performed using Affymetrix GeneAtlas Operating Software. The quality of the gene expression data was checked according to the quality control criteria provided by the software. The obtained CEL files were imported into the downstream data analysis software. All analyses were performed using BioConductor software based on the statistical R programming language. For background correction, normalization, and summation of raw data, the Robust Multiarray Averaging (RMA) algorithm implemented in the “affy” package of BioConductor was applied. Biological annotation was obtained from the BioConductor “oligo” package in which the annotated data frame object was merged with the normalized data set, leading to a complete gene data table. The selection criteria of significantly changed gene expression were based on an expression fold difference higher than |2|.

### 2.9. DAVID

Functional annotation clustering of differentially expressed genes was performed using DAVID (Database for Annotation, Visualization, and Integrated Discovery) [10]. Gene symbols for up- or downregulated genes from each of the compared groups were loaded into DAVID using the “RDAVIDWebService” BioConductor package. Functional annotation chats generated by DAVID with overrepresented gene annotations are shown as bubble plots from the BACA BioConductor package (https://cran.r-project.org/web/packages/BACA/BACA.pdf). Bubble plots were generated with the following criteria: *p* value < 0.5, adjusted method = Benjamini, and minimal number of genes per group = 5. Groups of genes fulfilling the mentioned criteria are presented in a graph in which the bubble size indicates the number of genes represented in the corresponding annotation and the condition of these genes in terms of their down- and upregulation.

### 2.10. Gene Set Enrichment Analysis (GSEA)

GSEA is a computational method that is used for testing a priori-defined gene sets (GO terms, pathways) for their association with one of the two compared biological groups. The method utilizes the Kolmogorov-Smirnov (K-S) statistical test to identify significantly enriched or depleted groups of genes [11]. GSEA analysis has been conducted using the GSEA Java Desktop Application from the Broad Institute (http://software.broadinstitute.org/gsea/index.jsp). Normalized data from all genes were transformed into an appropriate format and imported into the application. Then, a predefined gene set database was selected from the Molecular Signatures Database (MsigDB) [12]. Genes belonging to the selected set were ranked according to the difference in their expression level using the signal-to-noise ratio with 1000 permutations. By walking down the ranked list of genes, the enrichment score (ES) was calculated for each selected gene set. This procedure was performed using a sum statistic when a gene was present in the gene set, and the calculation was decreased when it was not [12]. Enrichment scores were normalized by their gene set size, and false-positive findings were corrected by FDR. Significant gene sets were considered to be those with an adjusted nominal *p* value < 0.01 and FRD *q* value < 25%.

### 2.11. Statistical Methods

The significance of the differences between experimental groups was assessed by the Mann–Whitney *U* test. *p* < 0.05 was considered statistically significant. The data are presented as the mean ± standard deviation (SD). Benjamini and Hochberg multiple testing correction was applied to correct for false-positive results in the microarray analysis.

## 3. Results

### 3.1. Functional and Morphological Evaluation of Aged Retinas from C3−/− and WT Mice

**Figure 1:**
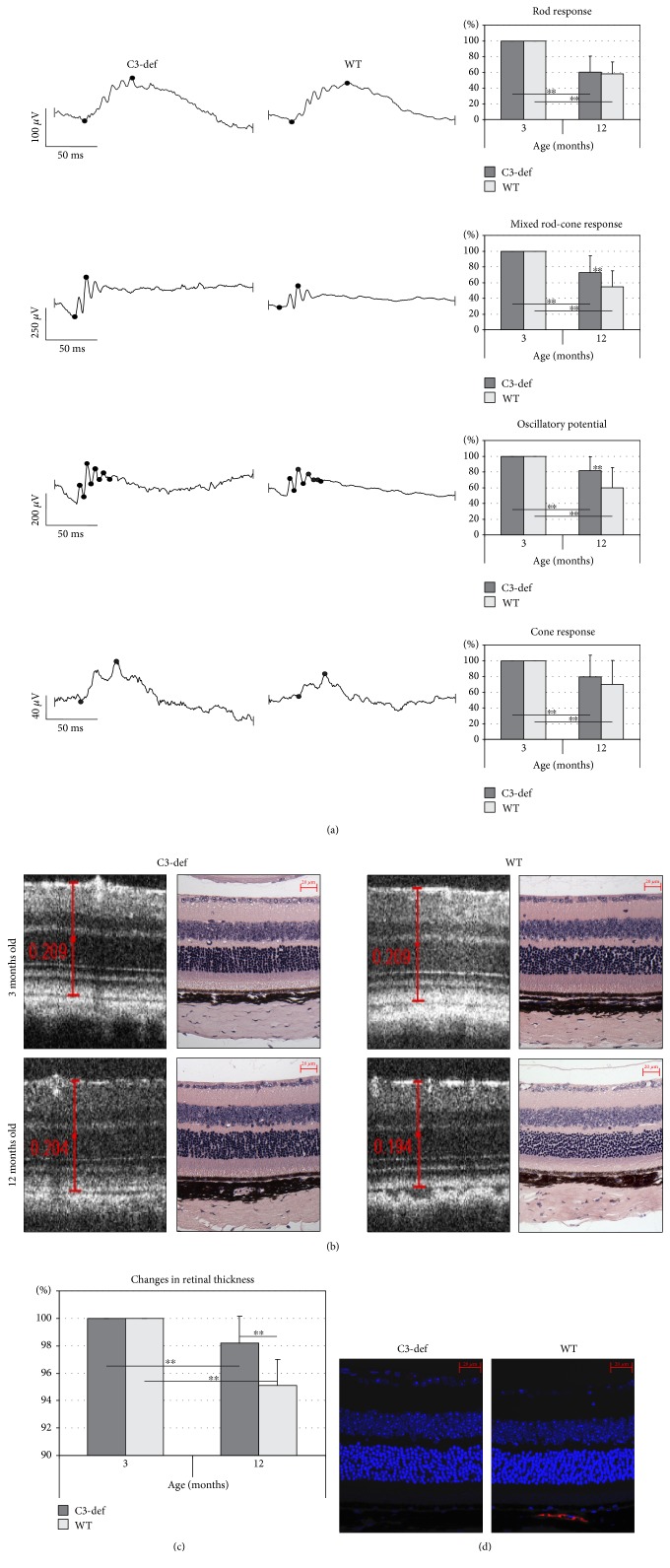
Functional and morphological evaluation of aged retinas from C3−/− and WT mice. (a) The representative rod, mixed rod-cone, and cone responses, as well as oscillatory potentials (OPs), recorded from 12-month-old C3-deficient (C3-def) and WT mice are shown. Changes in the retinal bioelectrical response during aging are presented as a percentage value calculated for each individual 1-year-old mice in reference to the b-wave amplitude measurements obtained from 3-month-old mice (considered 100%). The results are shown as mean ± SD (*n* = 10 mice per group). The ERG recordings at 12 months of age were reduced in both C3−/− and WT mice (^∗∗^*p* < 0.01 for scotopic and photopic conditions); however, when analyzing mixed rod-cone responses and OPs, the b-wave amplitudes in C3-deficient mice were significantly higher (^∗∗^*p* < 0.01) compared to age-matched WT animals. (b) The representative in vivo SD-OCT retinal scans and H&E-stained images of 3- and 12-month-old C3-def and WT mice are shown. The scale bar is 20 *μ*m. (c) Spectral domain optical coherence tomography was used to assess the changes in retinal morphology and thickness over time. Changes in retinal thickness are presented as a percentage value calculated for each individual 1-year-old mice in reference to measurements obtained from 3-month-old mice (considered 100%). The results are shown as mean ± SD (*n* = 10 mice per group). Aged retinas of both C3-def and WT mice showed the signs of declined thickness (^∗∗^*p* < 0.01); however, this process was more profound in WT mice (^∗∗^*p* < 0.01). (d) Immunofluorescence analysis of complement component C3 in aged WT retinas revealed deposition of the protein in the sub-RPE region. The sections from 12-month-old C3-deficient animals served as a staining control. The scale bar is 20 *μ*m.

To assess whether complement component C3 deficiency influenced the physiological function of aged retinas, we examined the retinal bioelectrical response in 3- and 12-month-old C3-deficient (C3−/−) and control (wild type (WT)) mice. The results are summarized in Figure 1(a). The ERG recordings, under both scotopic and photopic conditions, in C3−/− and WT mice at 12 months of age, were significantly lower (*p* < 0.01) than those observed in 3-month-old gender-matched animals. When comparing bioelectrical responses of the 12-month-old C3-deficient and WT mice, we found considerable differences in b-wave amplitudes between the groups of animals. In C3−/− mice, the mixed rod-cone response was significantly higher (*p* < 0.01) compared with that of the age-matched controls (72.81 ± 21.13% versus 54.78 ± 20.11%, resp., in reference to b-wave amplitude measurements obtained from 3-month-old gender-matched mice, mean ± SD, *n* = 10 mice per group). Similarly, the oscillatory potentials, which are thought to reflect the bioactivity initiated by amacrine cells in the inner retina, were considerably higher in C3−/− than in WT mice (81.54 ± 17.54% and 59.63 ± 26.04%, resp., *p* < 0.01). The differences of rod- and cone-driven response between C3−/− and WT animals at 12 months of age were not statistically significant. These results may suggest that C3 deficiency affects the function of photoreceptors.

Consequently, to evaluate age-related changes in retinal morphology and thickness, we performed spectral domain optical coherence tomography (SD-OCT) in 12-month-old C3-deficient and WT mice. The results are summarized in Figures 1(b) and 1(c). In both C3−/− and WT mice, the retinas at 12 months of age were thinner than those in the same mice at 3 months of age. When comparing the retinal thickness of the 12-month-old mice in both groups, the retinas of C3-deficient mice were found to be significantly (*p* < 0.01) thicker than those of the age-matched WT controls. These data provide evidence that the aged retinas of both C3−/− and WT mice exhibit age-related signs of reduced thickness; however, this process was significantly slower in C3-deficient mice.

To examine complement activation in aged WT mouse retinas, the immunofluorescence analysis of component C3 was applied. The sections from 12-month-old C3-deficient animals served as a staining control. We found depositions of C3 protein in the sub-RPE region (Figure 1(d)). This result suggests that complement activation may play a role in the process of natural retinal aging.

### 3.2. Oxidative Stress and Apoptosis Analysis in Aged Retinas from C3−/− and WT Mice

**Figure 2:**
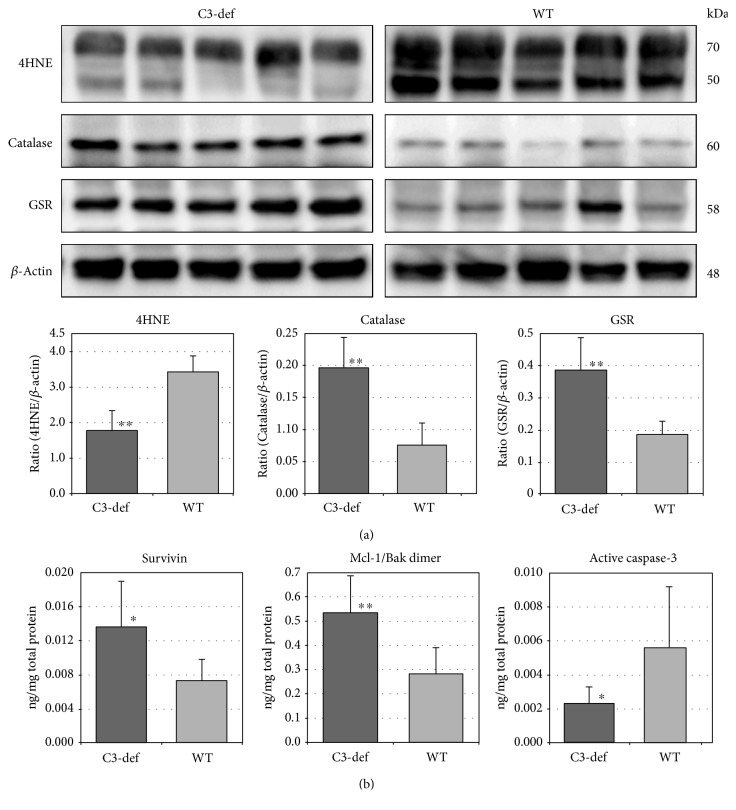
Evaluation of oxidative stress markers and apoptotic activity in the retinas of aged C3-deficient and WT mice. (a) Western blot and densitometry analysis of 4HNE, catalase, glutathione reductase (GSR), and *β*-actin, which served as an internal control. Immunoblot images are representative of three independent experiments yielding similar results. The protein expression level of the 4HNE, catalase, and GSR was quantified in comparison to that of *β*-actin and shown as mean ± SD (*n* = 14 eyes per group). 4HNE-modified proteins, an oxidative stress marker, were detected in both 12-month-old C3-def and WT mice, although the 4HNE/*β*-actin ratio was significantly lower in the retinas collected from C3-def mice (^∗∗^*p* < 0.01). Moreover, catalase and GSR levels, which represent antioxidant enzymes, were considerably higher in C3-deficient mice (^∗∗^*p* < 0.01). (b) Luminex multiplex fluorescent bead-based immunoassay was used to evaluate protein expression level of survivin, Mcl-1/Bak dimer, and active caspase-3 in retinal lysates. To standardize the final concentration values, the obtained data was normalized to the total protein concentration. The results are shown as mean ± SD (*n* = 14 eyes per group). C3-def mice demonstrated higher rates of survivin and Mcl-1/Bak dimer (^∗∗^*p* < 0.01), which represent antiapoptotic proteins (^∗^*p* < 0.05). The rates of active caspase-3 were considerably lower in C3-def mice (^∗^*p* < 0.05) as compared to age-matched control mice.

To determine a link between oxidative stress and the complement system, we performed expression analysis of selected proteins, including 4HNE (cellular oxidative stress marker), catalase (CAT), and glutathione reductase (GSR), in retinas collected from 12-month-old C3−/− mice and their WT counterparts. The results are summarized in Figure 2(a). We found that the 4HNE/*β*-actin ratio was significantly lower in retinas collected from C3−/− mice (*n* = 14 eyes per group, *p* < 0.01). Moreover, CAT and GSR, which represent antioxidative enzymes, were expressed at significantly higher levels (*p* < 0.01) in C3−/− mice. The above results suggest that aged retinas from old C3−/− mice possess higher antioxidant enzyme activities than do the retinas of their age-matched counterparts and thus are less susceptible to the local development of oxidative stress in retinal tissue.

Next, to assess apoptosis as cell death-related processes, the Luminex multiplex fluorescent bead-based immunoassay was used to evaluate the expression levels of selected proteins associated with pro- and antiapoptotic cell signaling, including survivin, Mcl-1/Bak dimer, and the active form of caspase-3 in retinal lysates from both 12-month-old C3−/− and WT mice (*n* = 14 eyes per group). As shown in Figure 2(b), the C3−/− mice demonstrated higher expression levels of survivin (*p* < 0.01) and Mcl-1/Bak dimer (*p* < 0.05), which represent antiapoptotic proteins. In contrast, the expression of proapoptotic active caspase-3 was significantly lower in C3−/− mice (*p* < 0.05) than in WT mice.

### 3.3. Clearance of Modified or Misfolded Proteins in Aged Retinas from C3−/− and WT Mice

**Figure 3:**
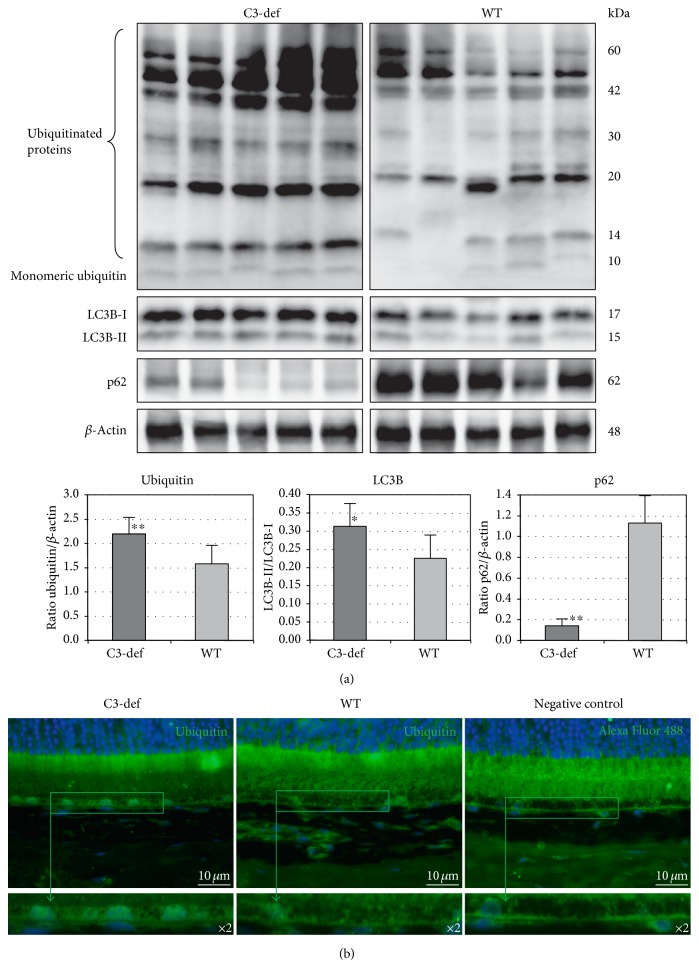
Evaluation of the autophagy markers in the retinas of aged C3-deficient and WT mice. (a) Western blot and densitometry analysis of ubiquitin, LC3B, p62, and *β*-actin, which served as an internal control. Immunoblot images are representative of three independent experiments yielding similar results. The protein expression level of the ubiquitin and p62 was quantified in comparison to that of *β*-actin. The LC3B protein level is presented as a LC3B-II/LC3B-I ratio. The results are shown as mean ± SD (*n* = 14 eyes per group). Western blot analysis in the retinas collected from C3-def mice demonstrates strong expression of the ubiquitinated proteins (^∗∗^*p* < 0.01) and higher LC3B-II/I ratio (^∗^*p* < 0.05) together with a decrease in p62 level (^∗∗^*p* < 0.01), compared to that from age-matched control mouse retinas. (b) Immunofluorescence staining of ubiquitin protein in the retinas of the 12-month-old C3-def and WT mice confirms the decreased expression of ubiquitin in RPE cells of WT mice. The scale bar is 10 *μ*m.

To examine the efficiency of age-related protein degradation mechanisms in retinal cells, we analyzed the expression of selected proteins involved in ubiquitination and autophagy, such as ubiquitin, LC3B-I, LC3B-II, and p62 from 12-month-old C3−/− and WT mice. The results are summarized in Figure 3. Since the conversion of LC3B-I to LC3B-II correlates well with the number of autophagosomes, the ratio of LC3B-II to LC3B-I was used as an indicator of autophagic activity. Accordingly, ubiquitin-binding p62 protein serves as a marker for the cargo subjected to autophagosomal degradation. As presented in Figure 3(a), we documented a significantly higher LC3B-II/I ratio (*p* < 0.05) together with a decreased expression of p62 protein (*p* < 0.01) in C3-deficient mice compared with the age-matched controls. Indeed, the Western blot analysis detected stronger bands of ubiquitin-tagged proteins in retinas collected from aged C3−/− mice (*n* = 14 eyes per group). Immunofluorescence staining of collected retinas confirmed the localization of ubiquitinated protein conjugates in the RPE layer (Figure 3(b)). Taken together, these observations imply that a complement system deficiency induces the cellular clearance of proteins through autophagy mechanisms in aged retinas.

### 3.4. Differential Gene Expression Profile

To identify molecular changes that are potentially responsible for the phenotype associated with the absence of complement component C3 in aged retina, we performed a detailed comparison of the transcriptional profiles of retinas isolated from 12-month-old C3-knockout and WT mice. Quantitative microarray analysis of RNA isolated from the collected retinas allowed the identification of 292 gene transcripts that showed at least a 2-fold significant difference in expression levels between the analyzed groups. Among them, 115 genes were overexpressed between 2- and 8.13-fold, whereas 177 genes were downregulated in a range from 2- to 25.95-fold in C3−/− mice compared with the age-matched WT counterparts. The top 25 up- and downregulated genes in aged C3-deficient mice are listed in Table 1. Within the group of overexpressed genes, the highest level of transcriptional activation was shown by enzymes that ensure vascular integrity, that is, serpin peptidase inhibitor and clade E (*Serpine3*), and the detoxification of metabolic products or environmental pollutants, that is, aldo-keto reductase family-1, member E1 (*Akr1e1*). Moreover, C3-knockout mice demonstrated higher expression levels of genes involved in maintaining the physiological function of the neural retina: abhydrolase domain containing 14A (*Abhd14a*); complexin 4 (*Cplx4*); cGMP specific-phosphodiesterase 6C (*Pde6c*); neurofilament (*Nefl*); aldehyde dehydrogenase family 1, subfamily A1 (*Aldh1a1*); retinitis pigmentosa GTPase regulator interacting protein-1 (*Rpgrip1*); and regulator of G protein signaling 9 binding protein (*Rgs9bp*). The most downregulated genes in retinas from C3-deficient mice were those associated with innate and adaptive immunity: regenerating islet-derived 3 *γ* (*Reg3g*), proplatelet basic protein (*Ppbp*), defensin *β*6 (*Defb6*), lactotransferrin (*Ltf*), mucin 5B (*Muc5b*), and a large group of genes coding basic structural units of immunoglobulins—*Ighj4*, *Igkv4-53*, *Igh-VJ558*, *Ighj1*, *Igj*, *Igkj1*, *Ighg2b*, *Ighj3*, *Ighg1*, and *Igkv1-110*.

**Figure 4:**
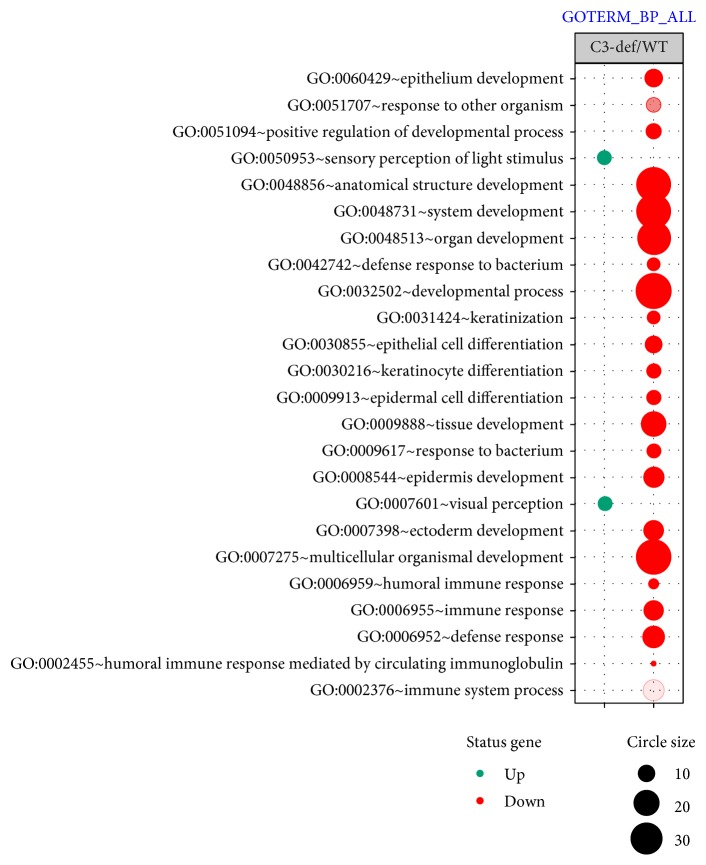
Overrepresented biological process assigned according to Gene Ontology (GO) classification in the retinas of aged C3-deficient mice compared to WT mice. Groups of genes fulfilling criteria: adjusted *p* value < 0.05, method = Benjamini, and minimum number of genes per group = 5, are presented in a graph, where bubble size indicates the number of genes represented in corresponding annotation and the condition of these genes in terms of their up- and downregulation.

Furthermore, up- and downregulated genes were assigned to specific biological processes according to the Gene Ontology (GO) classification. The bubble diagram illustrating overrepresented terms is shown in Figure 4. Analysis of functional annotations identified two biological processes that were significantly upregulated in C3-knockout animals: visual perception and sensory perception of light stimulus. These processes are represented in our study by several upregulated genes: *Gucy2f*, *Ppt1*, *Bbs2*, *Crb1*, *Gabrr2*, *Rgs9bp*, *Rpgrip*1, and *Pde6c*, which are essential for photoreceptor-based light-dependent signal transformation and transduction. In contrast, among the biological processes that were inhibited in C3−/− mice were those such as the immune response, defense response, humoral immune response, humoral immune response mediated by circulating immunoglobulin, and immune system process regulation. Further analysis of the GO functional annotations also revealed other general terms that were of interest. It should be emphasized that the GO database is still in the development stage and is far from being complete. Moreover, it is composed of some general as well as specific categories with a similar meaning; therefore, a single gene may be mapped to several GO terms and may be counted more than once [13].

**Figure 5:**
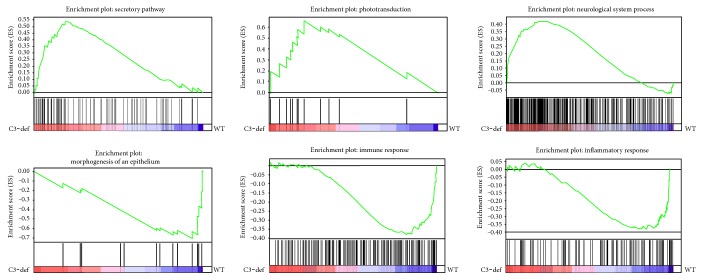
Gene set enrichment analysis (GSEA) of the retinas from aged C3-deficient mice compared to WT mice. Diagrams represent selected gene sets enriched in C3-deficient mice (secretory pathway, phototransduction, and neurological system process) and WT control animals (morphogenesis of an epithelium, immune response, and inflammatory response).

To more closely examine the biological influence of C3 deficiency on the aged retina, we performed gene set enrichment analysis (GSEA) to identify pathways that were altered in 12-month-old C3−/− mice compared with WT animals. GSEA detected 26 gene sets that were enriched in mouse knockouts for the C3 gene. Most of these were involved in neurological processes, such as the secretory pathway (normalized enrichment score (NES) = 2.02), synaptic transmission (1.98), transmission of nerve impulses (1.96), neurological system processes (1.90), synaptogenesis (1.75), phototransduction (1.70), nervous system development (1.67), synapse organization and biogenesis (1.66), central nervous system development (1.62), and sensory perception (1.61). Simultaneously, 29 gene sets were negatively correlated to C3−/− mice. Among them, the two largest groups were represented by gene sets associated with the immune system: response to bacterium (−2.12), defense response (−1.98), regulation of the immune response (−1.76), regulation of immune system processes (−1.68), and immune system processes (–1.61), and inflammation: acute inflammatory response (−2.05), inflammatory response (−1.76), cytokine- and chemokine-mediated signaling pathway (−1.73). Representative diagrams of the enriched gene sets are shown in Figure 5. A complete list of gene sets enriched in both aged C3−/− and WT mice is presented in Table 2.

## 4. Discussion

There is a general consensus that cumulative oxidative damage is responsible for aging and may therefore play an important role in the pathogenesis of retinal degeneration [14]. The retina is constantly challenged by excessive formation of ROS due to the high metabolic rate (i), high oxygen consumption (ii), long periods of exposure to short-wavelength light (iii), high concentration of polyunsaturated fatty acids (PUFAs) (iv), and phagocytic activity of RPE cells, which is accompanied by a respiratory burst—a rapid release of superoxide radical and hydrogen peroxide (v) [14–16]. Several studies have documented that the expression of intrinsic antioxidants, including catalase and glutathione reductase, has been shown to decline with age [17–19] and in AMD patients [17, 20]. In our study, we found that 12-month-old C3−/− mice exhibited lower levels of 4HNE-modified proteins—a marker of oxidative damage—compared with age-matched WT mice. Moreover, the expression of two antioxidant enzymes, catalase and glutathione reductase, was significantly increased in aged C3−/− mice. Our findings suggest that CS may act as a mediator of oxidative stress and local tissue injury. Indeed, a number of studies suggest that prolonged oxidative stress may cause retinal damage through a local dysregulation of the complement system [21]. Human ARPE-19 cells exposed to low-grade oxidative stress exhibited markedly impaired secretion of several complement regulators, including membrane-bound (DAF, MCP, and CD59) and fluid-phase (CFH) molecules [22–24], leading to uncontrolled complement activation.

It has also been proposed that protein modification by 4HNE is an early event that precedes photoreceptor cell apoptosis [25]. Herein, we found that the level of active caspase-3, a hallmark of the ongoing irreversible phase of apoptosis, was significantly lower in retinas from aged C3−/− mice compared with control animals and corresponded to significantly lower levels of 4HNE in retinas from old C3−/− mice. In addition, the levels of two other antiapoptotic proteins, survivin and Mcl-1/Bak dimer, were upregulated in C3-deficient animals. We infer that in C3−/− mice, the naturally lower levels of oxidative stress may explain the decreased age-related thinning of the retina observed in these animals as determined by OCT. Furthermore, C3-knockout mice appeared to be potentially protected from apoptosis by generating higher levels of antiapoptotic proteins such as survivin and Mcl-1/Bak dimer.

In an attempt to characterize the mechanisms underlying the beneficial effects of C3 deficiency, we analyzed the activity of autophagy and proteasomal cleansing in retinal cells and found that the absence of active C3 had a significant effect on the quality of this process. It is widely accepted that dysregulation of the UPP and autophagy processes is associated with a detrimental accumulation of aggregated proteins, which further increases the oxidative burden and can lead to the development of retinal degenerative disorders [4, 26, 27]. The UPP system is responsible for the degradation of 80%–90% of proteins, mostly short-lived, oxidatively modified, or damaged proteins [28, 29]. Autophagy is specialized to degrade aggregated proteins and cellular organelles [30]. The UPP and autophagy were previously regarded as two independent processes; however, it has recently been suggested that both cooperate with each other because ubiquitinylation may target substrates for degradation via both pathways [30, 31]. It has been documented that abundant oxidative stress negatively affects the autophagy process. Current data indicate that Nrf2 transcription factor, which is a key regulator of the oxidative stress defense system, interacts closely with the autophagy process via p62 protein [32–34]. Nrf2-knockout mice developed age-dependent degeneration of the RPE and choriocapillaries, spontaneous neovascularization, and deposits of inflammatory proteins in the sub-RPE space due to impaired autophagy and proteasomal cleansing processes. Moreover, in the RPE and Bruch's membrane of Nrf2-deficient mice, an age-dependent increase in immunoreactivity of C3d (a marker of complement activation), vitronectin (complement S-protein), and 3-nitrotyrosine (a marker of oxidatively damaged proteins) was observed [32]. The ubiquitin-binding p62/SQSTM1 (p62) protein serves as a marker for the cargo that is subjected to autophagosomal degradation [35]. Classically, the p62 level is expected to decrease due to degradation during the active autophagic process, and the increased expression of p62 indicates impaired autophagosomal degradation [36]. Indeed, retina samples from AMD patients showed an accumulation of p62 [37]. Another protein that is crucial for autophagy, LC3B-I, is found in preautophagosomal structures and converted to the LC3B-II form during the autophagosome maturation process. Finally, the mature autophagosomes are degraded by lysosomal hydrolytic enzymes [38]. Our group demonstrated a reduced expression of p62 and an elevated expression of the LC3B-II/LC3B-I ratio in retinas from aged C3−/− compared with WT mice. This result may indicate the good quality of the ongoing process of autophagosome formation in C3−/− mice and the successful packaging of waste material for autophagic degradation. The use of anti-ubiquitin antibody to monitor the localization of ubiquitin-positive cells further confirmed the Western blotting results. The ubiquitinated protein aggregate signal was higher in aged C3−/− retinal samples than in the WT controls and was mainly localized in the RPE layer. This finding reveals that C3 deficiency increases the amount of aggregate-prone proteins that undergo autophagy cleansing. Overall, our results indicate that CS suppression might facilitate the intracellular autophagic flux, as evidenced by the low levels of p62 together with high LC3B-II in the aged retinas.

Our findings support the notion that the ameliorative effects of C3 deficiency result from the compensatory ability to modulate the expression of several genes and proteins, which are components of signal transduction pathways that are important for retinal cell survival and function. Likewise, we were able to detect profound changes in the global gene expression profile using a comprehensive RNA microarray. Within the group of overexpressed genes in aged C3−/− animals, the highest levels of transcriptional activation were shown by *Serpine3* (8.13-fold), *Akr1e1* (4.41-fold), *Cplx4* (3.33-fold), *Pde6c* (2.59-fold), *Rpgrip* (2.54-fold), and *Rgs9bp* (2.50-fold). The *Serpine3* gene encodes an extracellular protein that belongs to the protease inhibitor family with activity toward thrombin, trypsin, and urokinase. It promotes neurite extension by inhibiting thrombin, and it possesses antiangiogenic properties [39]. *Akr1e1* is a member of the aldo-keto reductase family, which has cytoprotective effects against oxidative stress [40, 41]. Complexin 4 (*Cplx*4) is expressed mainly in photoreceptor and bipolar cell synapses [42]. Members of this protein family are responsible for the regulation of vesicle fusion and transmitter release, thus acting as a mediator of visual signal transmission [43]. *Pde6c* and *Rgs9bp* are both associated with normal phototransduction processes in cones and rods, respectively [44]. Retinitis pigmentosa-GTPase regulator-interacting protein (*Rpgrip*) has an important role in photoreceptor connecting cilia, helping to maintain correct photoreceptor function [45]. Based on the Gene Ontology classification of the biological processes, we identified two pathways that were significantly upregulated in aged C3-knockout mice: visual perception and sensory perception of light stimulus. Moreover, in addition to the results obtained using the GO database, GSEA detected 26 gene sets that were enriched in mice lacking the C3 gene, some of which were involved in neurological processes such as the secretory pathway, synaptic transmission, transmission of nerve impulses, neurological system processes, synaptogenesis, phototransduction, nervous system development, synapse organization and biogenesis, central nervous system development, and sensory perception. Of note, complement C3 naturally mediates immune-related activities, but it also facilitates other processes such as synapse elimination in the developing visual system of young mice [46]. However, in the aged brain, C3 contributes to region-specific synapse and neuron loss [47].

Furthermore, since component C3 plays a potent role in the host defense system, the most downregulated genes in aged C3-deficient mice were those associated with innate and adaptive immunity. These results are consistent with previous reports that C3-deficient subjects are more susceptible to microbial infections [48]. According to the GO classification, biological processes that were mostly inhibited in aged C3−/− mice included the humoral immune response, immune response, defense response, humoral immune response mediated by circulating immunoglobulin, and immune system process. Simultaneously, GSEA allowed us to identify 29 gene sets that were negatively correlated to aged C3-deficient mice. Among them, the largest two groups are represented by gene sets associated with inflammation: acute inflammatory response, inflammatory response, cytokine- and chemokine-mediated signaling pathway, and the immune system: response to bacterium, defense response, regulation of immune response, regulation of immune system process, and immune system process. Importantly, annotations involved in epithelial development and epithelial cell differentiation were significantly upregulated in WT mice, probably due to active mechanisms for countering the negative effects of oxidative stress on the RPE cells.

## 5. Conclusions

In summary, our study was designed to investigate the influence of complement component C3 global depletion on the biological structure and function of the aged retina. Aging is a significant risk factor for retinal degenerative diseases, and our collective data implicate C3 as potentially playing a role in the process of retinal degeneration. Importantly, the absence of active C3 resulted in decreased proapoptotic caspase-3 activity; elevated levels of antioxidant enzymes (catalase and glutathione reductase), autophagy-related ubiquitin, and cytosolic fraction of LC3B-II; and increased expression of several gene sets associated with maintaining physiological functions of the neural retina. All these changes potentially suggest that C3 inhibition may be protective against retinal degeneration. Our findings shed light on mechanisms of age-related retinal alterations identifying C3 as a potential therapeutic target for retinal aging. In particular, targeting the complement system may be promising for future therapeutic strategies in AMD disorders. Nevertheless, our observations were obtained in a model of systemic genetically depleted C3 in the whole organism and not selectively in ocular tissues. Therefore, further investigations should focus on targeted inhibition of C3 in the retina in various disease models to better understand the biological effects of C3 deficiency.

## Figures and Tables

**Table 1:** List of top 25 up- and downregulated genes in 12-month-old C3-deficient mice. Genes are ranked according to the expression fold difference—the gene from the top of the list is the most upregulated and the gene from the bottom of the list is the most downregulated in C3-deficient mice compared to control animals.

Direction in C3-def	Gene symbol	Gene name	Probe ID	Entrez Gene ID	Fold change
Up	Serpine3	Serpin peptidase inhibitor, clade E (nexin, plasminogen inhibitor)	17301155	319433	8.13
Up	Akr1e1	Aldo-keto reductase family 1, member E1	17290301	56043	4.41
Up	Edv	Endogenous sequence related to the Duplan murine retrovirus	17548311	13623	4.28
Up	Rpl29	Ribosomal protein 29	17521204	19944	4.23
Up	Abhd14a	Abhydrolase domain containing 14A	17530686	68644	4.02
Up	Gatc	Glutamyl-tRNA (Gln) amidotransferase, subunit C homolog	17451721	384281	3.70
Up	Cplx4	Complexin 4	17355086	225644	3.33
Up	Uprt	Uracil phosphoribosylotransferase (FUR1) homolog	17537003	331487	3.07
Up	H2-T22	Histocompatibility 2, T region locus 22	17344593	15039	3.02
Up	Mzt1	Mitotic spindle organizing protein 1	17309065	76789	3.01
Up	Mpc1	Mitochondrial pyruvate carrier 1	17284037	55951	2.81
Up	Adam4	A disintegrin and metallopeptidase domain 4	17282343	11498	2.78
Up	Rab6b	RAB6B, member RAS oncogene family	17520922	270192	2.75
Up	Pcdhb18	Protocadherin beta 18	17349930	93889	2.67
Up	Cdh12	Cadherin 12	17310550	215654	2.65
Up	Fabp4	Fatty acid-binding protein 4, adipocyte	17404091	11770	2.59
Up	Pde6c	Phosphodiesterase 6C, cGMP specific, cone, alpha prime	17359118	110855	2.59
Up	Fam213a	Family with sequence similarity 213, member A	17305221	70564	2.59
Up	Nefl	Neurofilament, light peptide	17301582	18039	2.57
Up	Akap6	A kinase (PRKA) anchor protein 6	17275436	238161	2.57
Up	Aldh1a1	Aldehyde dehydrogenase family 1, subfamily A1	17358103	11668	2.55
Up	Hdac1	Histone deacytelase 1	17430397	433759	2.55
Up	Rpgrip1	Retinitis pigmentosa Gtpase regulator-interacting protein 1	17299715	77945	2.54
Up	Rgs9bp	Regulator of G protein signaling 9 binding protein	17489809	243923	2.50
Down	Il1f9	Interleukin 1 family, member 9	17367652	215257	−5.53
Down	Igkv1-110	Immunoglobulin kappa variable 1-110	17459338	381777	−5.64
Down	Muc5b	Mucin 5, subtype B, tracheobronchial	17485098	74180	−6.06
Down	Krt10	Keratin 10	17269064	16661	−6.11
Down	Krtdap	Keratinocyte differentiation-associated protein	17476557	64661	−6.39
Down	Sprr2f	Small proline-rich protein 2F	17399876	20760	−6.46
Down	Ighg1	Immunoglobulin heavy constant gamma 1 (G1m marker)	17284339	16017	−6.48
Down	Ighj3	Immunoglobulin heavy joining 3	17284356	777655	−7.50
Down	Ighg2b	Immunoglobulin heavy constant gamma 2B	17284334	16016	−7.73
Down	Igkj1	Immunoglobulin kappa joining 1	17459415	110759	−8.18
Down	Defb6	Defensin beta 6	17499602	116746	−8.34
Down	Igj	Immunoglobulin joining chain	17449447	16069	−8.53
Down	Cryba4	Crystalline, beta A4	17451195	12959	−8.55
Down	Ltf	Lactotransferrin	17522555	17002	−8.70
Down	Cnfn	Cornifelin	17487919	72383	−8.97
Down	Ighj1	Immunoglobulin heavy joining 1	17284360	777648	−10.53
Down	Klk13	Kallikrein-related peptidase 13	17477191	626834	−10.71
Down	Lgsn	Lengsin, lens protein with glutamate synthetase domain	17211533	266744	−11.56
Down	Lce1c	Late cornified envelope 1C	17399899	73719	−12.31
Down	Igh-VJ558	Immunoglobulin heavy chain (J558 family)	17284314	16061	−13.62
Down	Rps13	Ribosomal protein S13	17493182	68052	−14.76
Down	Igkv4-53	Immunoglobulin kappa variable 4-53	17459423	546213	−16.50
Down	Ighj4	Immunoglobulin heavy joining 4	17284354	777656	−18.58
Down	Ppbp	Proplatelet basic protein	17438963	57349	−21.02
Down	Reg3g	Regenerating islet-derived 3 gamma	17467973	19695	−25.95

**Table 2:** Top gene sets enriched in 12-month-old C3-deficient and WT mice. Gene sets are ranked according to the normalized enrichment score (NES)—the gene set from the top of the list is the most enriched in C3-def mice and the gene set from the bottom of the list is the most enriched in WT mice.

Direction in C3-def	Gene set	Size	ES	NES	NOM *p* value	FDR *q* value
Up	Secretory pathway	83	0.54	2.02	0.000	0.014
Up	Synaptic transmission	164	0.48	1.98	0.000	0.021
Up	Transmission of nerve impulse	179	0.47	1.96	0.000	0.021
Up	Neurological system process	359	0.42	1.90	0.000	0.038
Up	Nuclear export	32	0.60	1.84	0.000	0.076
Up	Golgi vesicle transport	48	0.54	1.81	0.000	0.097
Up	Brain development	47	0.53	1.80	0.000	0.090
Up	Secretion by cell	114	0.45	1.79	0.000	0.101
Up	Exocytosis	24	0.60	1.76	0.007	0.126
Up	Synaptogenesis	15	0.66	1.75	0.009	0.115
Up	RNA export from nucleus	19	0.63	1.73	0.003	0.131
Up	Regulation of cell-cell adhesion	9	0.77	1.72	0.007	0.135
Up	Microtubule-based movement	16	0.64	1.71	0.007	0.150
Up	Phototransduction	13	0.66	1.70	0.007	0.154
Up	Nervous system development	352	0.37	1.67	0.000	0.187
Up	Chromosome organization and biogenesis	118	0.42	1.67	0.000	0.184
Up	NLS-bearing substrate import into nucleus	12	0.67	1.67	0.017	0.175
Up	Synapse organization and biogenesis	20	0.60	1.66	0.022	0.174
Up	Establishment of cellular localization	343	0.36	1.63	0.000	0.216
Up	Central nervous system development	116	0.41	1.62	0.000	0.220
Up	Intracellular transport	272	0.37	1.62	0.001	0.196
Up	Cellular localization	360	0.36	1.62	0.000	0.194
Up	Sensory perception	181	0.38	1.61	0.001	0.206
Up	Vesicle-mediated transport	189	0.38	1.58	0.000	0.216
Up	Secretion	175	0.38	1.57	0.000	0.213
Up	Organelle organization and biogenesis	453	0.33	1.53	0.000	0.245
Down	Translation	163	−0.34	−1.51	0.003	0.241
Down	Response to external stimulus	297	−0.33	−1.58	0.000	0.216
Down	Immune system process	304	−0.34	−1.61	0.000	0.194
Down	Regulation of protein modification process	41	−0.47	−1.61	0.007	0.200
Down	Positive regulation of signal transduction	123	−0.38	−1.63	0.000	0.181
Down	Regulation of immune system process	64	−0.43	−1.68	0.003	0.158
Down	Response to wounding	182	−0.38	−1.68	0.000	0.158
Down	Response to biotic stimulus	107	−0.41	−1.69	0.000	0.155
Down	Regulation of protein amino acid phosphorylation	28	−0.54	−1.70	0.005	0.159
Down	Cytokine- and chemokine-mediated signaling pathway	22	−0.58	−1.73	0.009	0.127
Down	Developmental growth	11	−0.71	−1.75	0.007	0.113
Down	Regulation of protein import into nucleus	16	−0.64	−1.75	0.009	0.114
Down	Epithelial cell differentiation	9	−0.76	−1.76	0.010	0.115
Down	Immune response	214	−0.38	−1.76	0.000	0.120
Down	Inflammatory response	123	−0.42	−1.76	0.000	0.128
Down	Multiorganism process	134	−0.41	−1.76	0.000	0.134
Down	Regulation of immune response	32	−0.54	−1.76	0.005	0.143
Down	Regulation of phosphorylation	46	−0.51	−1.81	0.000	0.097
Down	Positive regulation of epithelial cell proliferation	10	−0.75	−1.84	0.000	0.077
Down	Morphogenesis of an epithelium	15	−0.71	−1.88	0.005	0.050
Down	Response to other organisms	72	−0.50	−1.94	0.000	0.023
Down	Tissue development	133	−0.47	−1.97	0.000	0.016
Down	Defense response	239	−0.43	−1.98	0.000	0.015
Down	Detection of biotic stimulus	10	−0.83	−2.04	0.000	0.010
Down	Acute inflammatory response	11	−0.85	−2.05	0.000	0.009
Down	Keratinocyte differentiation	14	−0.77	−2.08	0.000	0.005
Down	Response to bacterium	28	−0.67	−2.12	0.000	0.001
Down	Ectoderm development	76	−0.58	−2.23	0.000	0.001
Down	Epidermis development	67	−0.62	−2.37	0.000	0.000
